# 

**Published:** 1860-10

**Authors:** 


### CONTENTS.

### ORIGINAL COMMUNICATIONS.

PAGE.
ARTICLE I.—Abstract from the Minutes of Atlanta Medical Society 441
ARTICLE II.—Medical Topography of Wharton, Texas. By Rowan Green, M.D , Wharton, Texas, .......... 445

### SELECTIONS.

Experiments on the Recurrent Sensibility of the Anterior Roots of the Spinal Nerves .......... 449
Successful Operation for the Radical Cure of Hernia .......... 456
Cases Illustrative of the Influence of Relladonna .......... 459
On the Treatment of Tetanus by Liquor Potassæ .......... 464
The Oil of Turpentine in the Treatmen of Neuralgia, and particularly of Sciatica .......... 467
Veratrum Viride in Puerperal Mania .......... 470
On Veratrum Viride .......... 475
Gossypium Herbaceum (Cotton) .......... 479
On Anacahuite Wood .......... 485
Note on Anacahuite Wood, a reputed Remedy for Consumption .......... 486
Placenta Prævia; Treatment by the Caoutchouc Water Pessary .......... 490

### EDITORIAL AND MISCELLANEOUS.

The Generation of Insects in the Alimentary Canal .......... 494
Influenza .......... 495
Editorial Items .......... 496
Insanity .......... 497
Correspondence .......... 498
New Orleans Medical Times .......... 500
Items .......... 501
List of Payments, .......... 502

